# The association between perceived discrimination profiles and mental health among college students with hearing impairment in China: evidence from a latent profile analysis

**DOI:** 10.3389/fpubh.2026.1945174

**Published:** 2026-10-29

**Authors:** Jin Hu, Chaocao Yang, Songbai Xiang

**Affiliations:** 1 Sichuan Key Laboratory of Philosophy and Social Sciences for Language Intelligence in Special Education, Leshan, China; 2 College of Special Education, Leshan Normal University, Leshan, China; 3 College of Education, China West Normal University, Nanchong, China

**Keywords:** college students with hearing impairment, latent profile analysis, mental health, perceived discrimination, psychological stress

## Abstract

**Background:**

College students with hearing impairment are at increased risk of mental health problems due to communication barriers, limited social support, and adaptation challenges. Although perceived discrimination is closely related to mental health, less is known about whether subgroups characterized by different levels of perceived discrimination can be identified. This study aimed to identify perceived discrimination subgroups, compare mental health across subgroups, and examine demographic factors associated with subgroup membership.

**Methods:**

A cross-sectional survey was conducted among college students with hearing impairment recruited from 21 universities across 17 provinces and municipalities in China. Of the 1,151 questionnaires collected, 1,068 valid responses were included in the analysis, including 566 male students (53.0%; mean age = 20.13 years, SD = 0.90) and 502 female students (47.0%; mean age = 20.43 years, SD = 0.94). Participants completed a sociodemographic questionnaire, the six-item Perceived Discrimination Scale, and the 12-item General Health Questionnaire. Latent profile analysis was used to identify subgroups based on individual- and group-level perceived discrimination. The Bolck-Croon-Hagenaars method was applied to compare mental health problems across subgroups, and multinomial logistic regression was conducted to examine demographic factors associated with subgroup membership.

**Results:**

A three-class solution was selected as the most parsimonious and interpretable model, identifying subgroups characterized by low (28.6%), moderate (48.6%), and high (22.8%) levels of perceived discrimination. Individual- and group-level perceived discrimination increased in parallel across subgroups, indicating that the identified classes primarily reflected differences in the overall severity of perceived discrimination rather than qualitatively distinct configurations. Significant differences in subgroup membership were found by gender, peer type, and number of children in the family. Mental health problems differed significantly across the three subgroups, with the high perceived discrimination group reporting the most severe problems, followed by the moderate perceived discrimination group, and the low perceived discrimination group reporting the fewest.

**Conclusion:**

College students with hearing impairment can be differentiated into subgroups characterized by low, moderate, and high levels of perceived discrimination. These subgroups were associated with different levels of mental health problems, highlighting the potential value of identifying students with elevated perceived discrimination for targeted mental health support.

## Introduction

College students with hearing impairment often face substantial challenges in social interaction, including communication barriers, limited social support, identity-related difficulties, and adaptation-related stress. These challenges may place students with hearing impairment at increased risk of psychological distress and poor mental health outcomes (1, 2). As inclusive higher education expands, the mental health of students with hearing impairment and its associated determinants have become an increasingly important public health concern.

Perceived discrimination refers to the subjective experience of differential or unfair treatment based on one’s membership in a particular social group, as distinguished from objectively documented discrimination (3). College students with hearing impairment may encounter misunderstanding, prejudice, and exclusion because of differences in hearing and communication modes, particularly in predominantly hearing environments, and may therefore report greater perceived discrimination. According to the Rejection–Identification Model, perceived rejection by a dominant group may undermine psychological well-being, whereas identification with one’s own group may partially buffer this adverse effect (4). Previous studies have linked higher perceived discrimination among college students with hearing impairment to lower self-esteem (5), poorer social adaptation (6), lower achievement motivation (7), and weaker emotion regulation abilities (8). Together, these findings suggest that perceived discrimination may be an important psychosocial correlate of mental health problems in this population.

Existing research has identified several psychosocial factors relevant to perceived discrimination and mental health among students with hearing impairment. Perceived discrimination has been linked to poorer well-being among deaf and hard-of-hearing young people (9), whereas psychological resilience and adaptive coping may serve as protective resources among college students with disabilities (10). Social support, peer relationships, and school connectedness have also been associated with better psychosocial outcomes among deaf adolescents (11). In addition, evidence from identity-based interventions in other marginalized populations suggests that strengthening positive group identity may support more adaptive responses to discrimination-related experiences (12). Although these studies provide valuable insights, most have adopted variable-centered approaches that focus on average relationships across the full sample. Consequently, less attention has been paid to whether meaningful subgroups of college students with hearing impairment can be distinguished according to their levels of perceived discrimination.

College students with hearing impairment may vary substantially in their levels of perceived discrimination. Treating them as a homogeneous population may obscure meaningful differences in the severity of perceived discrimination and hinder the identification of subgroups at elevated risk. Latent profile analysis (LPA), a person-centered analytical approach, provides a useful means of identifying unobserved subgroups based on patterns of responses across discrimination indicators (13). Accordingly, the present study aimed to explore whether meaningful subgroups could be identified based on perceived discrimination indicators, compare mental health problems across these subgroups, and examine demographic factors associated with subgroup membership. The findings may inform the identification of students reporting elevated perceived discrimination and the development of more tailored mental health support in university settings.

## Materials and methods

### Participants

Data were collected from July 2024 to December 2025. Participants were recruited from 21 universities across 17 provinces and municipalities in eastern, central, western, southern, and northern China. These universities admit students with hearing impairment through separate entrance examinations for students with disabilities. Recruitment information and a link to the online questionnaire were distributed through university class WeChat groups and official class notices, and participation was voluntary. A total of 1,151 questionnaires were collected. Questionnaires were excluded if they met any of the following criteria: (1) more than 20% missing item responses (*n* = 23); (2) an abnormally short completion time (<120 s; *n* = 31); (3) patterned responding, such as selecting the same response option for all items (*n* = 18); or (4) an extreme standardized total perceived discrimination score (|*z*| > 3.29; *n* = 11). After these exclusions, 1,068 valid questionnaires were retained, accounting for 92.8% of the questionnaires collected.

Among the 1,068 participants, 566 were male (53.0%; *M*_age_ = 20.13, SD = 0.90) and 502 were female (47.0%; *M*_age_ = 20.43, SD = 0.94). In terms of academic year, 293 participants were freshmen (27.4%), 414 were sophomores (38.8%), 293 were juniors (27.4%), and 68 were seniors (6.4%). Regarding the degree of hearing loss, 628 participants were classified as having Grade I deafness (58.8%), 216 as having Grade II deafness (20.2%), 120 as having Grade I hard of hearing (11.2%), and 104 as having Grade II hard of hearing (9.7%). Hearing loss was classified according to the Chinese national standard *Classification and Grading Criteria of Disability* (GB/T 26341-2010), based on the pure-tone average (PTA) of the better ear at 500, 1000, 2000, and 4,000 Hz. Grade I, II, III, and IV hearing disability correspond to PTA values of >90, 81–90, 61–80, and 41–60 dB HL, respectively. Because the original audiometric records were not available, the specific testing conditions, including whether audiometric testing was conducted with or without hearing aids or cochlear implants, could not be confirmed. With respect to peer or close-friend type, 672 participants reported having deaf friends (62.9%), 309 reported having hearing friends (28.9%), and 87 reported having no close friends (8.1%). Regarding the number of children in the family, 273 participants came from one-child families (25.6%), 546 from two-child families (51.1%), 137 from three-child families (12.8%), and 112 from families with more than three children (10.5%).

This study was conducted in accordance with the Declaration of Helsinki and was approved by the Institutional Review Board of the Sichuan Provincial Key Laboratory of Philosophy and Social Science for Language Intelligence in Special Education (Approval No. KYLL2024-12-01). All participants provided informed consent before participation.

## Measures

### Mental health

The scale consists of 12 items rated on a 4-point Likert scale ranging from 1 (“not at all”) to 4 (“often”) (40). The Likert scoring method (1-2-3-4), rather than the traditional GHQ scoring method (0-0-1-1), was used. After reverse scoring Items 1, 3, 4, 7, 8, and 12, the item scores were summed to yield a total score ranging from 12 to 48, with higher scores indicating greater nonspecific psychological distress and mental health problems. The GHQ-12 is a screening measure of general psychological distress rather than a diagnostic instrument for psychiatric disorders or psychological crises. The GHQ-12 has been widely used in diverse populations and has demonstrated satisfactory reliability and validity.

#### Perceived discrimination

Perceived discrimination was assessed using the six-item Perceived Discrimination Scale developed by Shen et al. (14). The scale comprises two dimensions: individual perceived discrimination and group perceived discrimination, with three items in each dimension. Individual perceived discrimination was assessed using three items: S1, “I feel that I have been treated unfairly”; S2, “I feel that I am looked down upon by others”; and S3, “I feel that, compared with others, I have lost some opportunities.” Group perceived discrimination was assessed using three corresponding items adapted to the hearing-impairment context: S4, “Overall, other students with hearing impairment like me have been treated unfairly”; S5, “Overall, other students with hearing impairment like me have lost some opportunities”; and S6, “Overall, other students with hearing impairment like me are looked down upon.” All items were rated on a 5-point scale ranging from 1 (“not at all true”) to 5 (“completely true”), with higher scores indicating higher levels of perceived discrimination. In the present study, the scale demonstrated excellent internal consistency (Cronbach’s *α* = 0.94). For the LPA, the six items were labeled S1–S6, with S1–S3 representing individual perceived discrimination and S4–S6 representing group perceived discrimination. Confirmatory factor analysis showed acceptable model fit (*χ*^2^/d*f* = 6.59, RMSEA = 0.07, SRMR = 0.01, CFI = 0.99, TLI = 0.99), with standardized factor loadings ranging from 0.83 to 0.87. The composite reliability (CR) values for the two dimensions were 0.88 and 0.90, and the average variance extracted (AVE) values were 0.71 and 0.75, respectively, indicating good convergent validity. However, the correlation between the two latent factors was 0.99, indicating substantial overlap and limited discriminant validity between the two dimensions.

### Data analysis

Data analyses were conducted using Mplus 8.3 and SPSS 27.0. First, LPA was performed using the six item-level indicators of the Perceived Discrimination Scale, with S1–S3 assessing individual perceived discrimination and S4–S6 assessing group perceived discrimination. Several commonly used fit indices were considered in determining the final class solution (15, 16), including the Akaike Information Criterion (AIC), Bayesian Information Criterion (BIC), entropy, the Lo–Mendell–Rubin likelihood ratio test (LMR), and the bootstrap likelihood ratio test (BLRT). Lower AIC and BIC values indicate better model fit, whereas entropy values closer to 1 indicate greater classification accuracy. Significant LMR and BLRT results (*p* < 0.05) suggest that a model with *k* classes provides a better fit than a model with *k* − 1 classes. The final number of classes was determined by considering statistical fit, classification accuracy, class size, model parsimony, and substantive interpretability rather than relying on any single fit index (17–19).

Subsequently, analysis of variance (ANOVA) was used to examine differences in perceived discrimination dimensions across latent classes. The Bolck-Croon-Hagenaars (BCH) method was applied to compare mental health problems across the latent classes. To examine the associations between demographic factors and latent class membership while accounting for classification uncertainty, the R3STEP procedure implemented in Mplus was used. This three-step approach incorporates classification error into the estimation of associations between covariates and latent class membership, thereby reducing potential bias arising from uncertainty in class assignment. As a sensitivity analysis, hierarchical multiple linear regression was conducted to examine whether the association between perceived discrimination and mental health remained when perceived discrimination was treated as a continuous variable. Gender, peer type, number of children in the family, and family economic status were entered as covariates in Model 1, followed by the total perceived discrimination score in Model 2.

## Results

### Common method bias test

Because all data were collected from a single source, common method bias was assessed. A single-factor CFA model was specified, with all items loading onto one common latent factor. The model fit was poor (*χ*^2^/d*f* = 26.44, RMSEA = 0.154, SRMR = 0.024, CFI = 0.963, TLI = 0.930), suggesting that the one-factor model did not adequately fit the data. These results indicate that a single common method factor was insufficient to account for the covariance structure of the data. However, because all data were collected using self-report measures at the same time point, shared method variance cannot be completely ruled out. Therefore, the one-factor CFA results should be interpreted as supplementary diagnostic evidence rather than definitive evidence of the absence of common method bias.

### Correlation analysis

The results of the correlation analysis showed that overall perceived discrimination was significantly positively associated with both individual perceived discrimination and group perceived discrimination, with correlation coefficients ranging from 0.88 to 0.97. Furthermore, all dimensions of perceived discrimination were significantly positively associated with mental health problems, with correlation coefficients ranging from 0.45 to 0.47. Detailed results are shown in Table 1.

**Table 1:** Correlations among study variables.

Variable	*M* ± SD	1	2	3	4
1. Individual perceived discrimination	2.88 ± 1.02	1			
2. Group perceived discrimination	2.84 ± 1.02	0.88^**^	1		
3. Overall perceived discrimination	2.86 ± 0.99	0.97^**^	0.97^**^	1	
4. Mental health	2.29 ± 0.44	0.47^**^	0.45^**^	0.47^**^	1

*p < 0.05; **p < 0.01; ***p < 0.001. The same applies below.

### Model fit for perceived discrimination class solutions

Mplus 8.3 was used to conduct a LPA of perceived discrimination among college students with hearing impairment. Models with two to five latent classes were estimated sequentially, and the fit indices are presented in Table 2. Entropy values for all models exceeded 0.90, indicating high classification accuracy. The bootstrap likelihood ratio test (BLRT) was significant for the two-, three-, four-, and five-class models. Although the AIC, BIC, and adjusted BIC (aBIC) values continued to decrease as additional classes were added, model selection in LPA should not be determined by any single statistical criterion. Rather, the number of classes should be determined by considering multiple sources of evidence, including statistical fit, classification accuracy, class size, model parsimony, and substantive interpretability (18, 19). The rate of decline in the information criteria became less pronounced after the three-class solution, while the three-class model maintained high classification accuracy (Entropy = 0.933) and yielded three adequately sized classes, accounting for 28.6%, 48.6%, and 22.8% of the sample, respectively. In contrast, the five-class solution included one class comprising less than 5% of the total sample, which reduced the practical interpretability of the more complex solution. Although the four-class solution showed further improvement in the information criteria, improvements in statistical fit should be considered alongside increased model complexity and the substantive meaning of additional classes rather than being used as the sole basis for model selection (17, 19). Given the high correlation between the two dimensions represented by the six item-level indicators and the fact that the identified classes were primarily differentiated by the overall level of perceived discrimination, a more complex class solution may represent a finer subdivision of the same underlying severity continuum without necessarily providing additional substantively meaningful differentiation. Considering the balance among statistical fit, classification accuracy, class size, model parsimony, and substantive interpretability, the three-class solution was therefore selected as the most parsimonious and interpretable representation of the data for the purposes of the present study.

**Table 2:** Fit indices for the LPA of perceived discrimination.

Model	AIC	BIC	aBIC	Entropy	LMR (*p*)	BLRT (*p*)	Class probabilities
1	19769.797	19829.479	19791.365				
2	16580.247	16674.745	16614.397	0.929	<0.001	<0.001	0.34/0.66
3	15025.256	15154.568	15071.988	0.933	<0.001	<0.001	0.29/0.48/0.23
4	14572.129	14736.256	14631.442	0.923	<0.001	<0.001	0.11/0.22/0.45/0.22
5	14217.746	14416.688	14289.640	0.939	0.023	<0.001	0.11/0.21/0.04/0.41/0.22

As shown in Table 3, the posterior probabilities of class membership for the three latent classes (C1, C2, and C3) were 0.99, 0.97, and 0.95, respectively, indicating good classification reliability for the three-class solution. Based on this solution, the conditional mean estimates for each perceived discrimination item were obtained for the three classes, as illustrated in Figure 1. The classes were then named according to their score characteristics. Class 1 comprised 305 individuals (28.6%) and showed the lowest overall level of perceived discrimination, with all item scores below 2; it was therefore labeled the low perceived discrimination group. Class 2 comprised 519 individuals (48.6%), with item scores consistently falling between those of C1 and C3; it was therefore labeled the moderate perceived discrimination group. Class 3 comprised 244 individuals (22.8%) and showed the highest overall level of perceived discrimination; it was therefore labeled the high perceived discrimination group.

**Table 3:** Classification probabilities for the three-class solution.

Class	Probabilities		
	C1	C2	C3
C1	0.99	0.01	0.00
C2	0.02	0.97	0.02
C3	0.00	0.05	0.95

**Figure 1:**
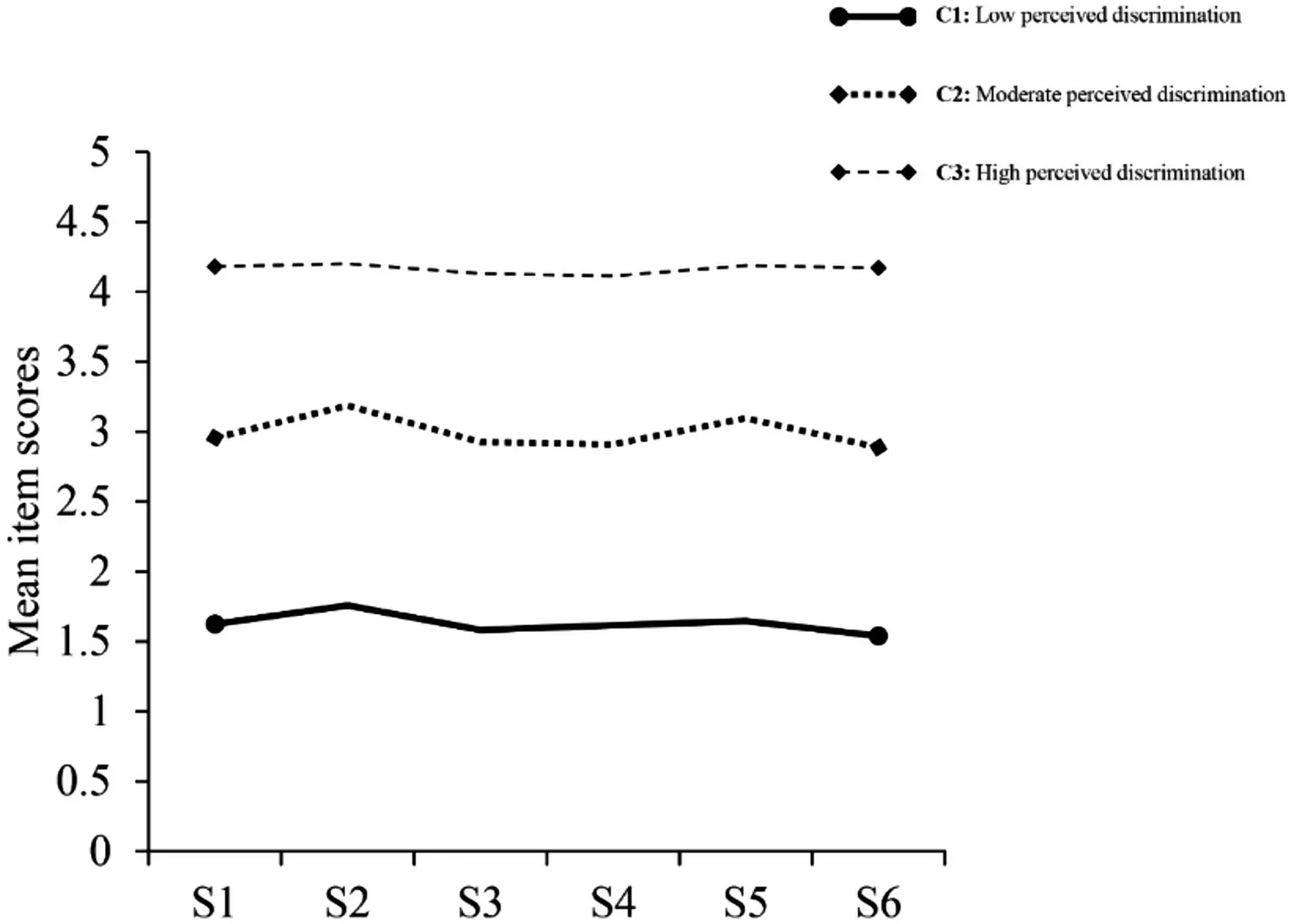
Distribution of perceived discrimination levels across the three latent classes among college students with hearing impairment.

Moreover, perceived discrimination scores differed significantly across the three severity-based subgroups for individual perceived discrimination, group perceived discrimination, and overall perceived discrimination (see Table 4), with *F* (2, 1,065) = 2108.16, *F* (2, 1,065) = 2302.88, and *F* (2, 1,065) = 3159.00, respectively; all *p* < 0.001. *Post hoc* comparisons further indicated that the high perceived discrimination group had the highest scores on individual perceived discrimination, group perceived discrimination, and overall perceived discrimination, followed by the moderate perceived discrimination group, while the low perceived discrimination group had the lowest scores. These results were consistent with the severity-based labeling of the three classes, with both discrimination indicators increasing progressively from the low to the high perceived discrimination subgroup.

**Table 4:** Differences in perceived discrimination dimensions across the three latent classes.

Dimension of perceived discrimination	Latent class			*F*	*η* ^2^	*Post hoc* comparison
	C1	C2	C3			
1. Individual perceived discrimination	1.64 ± 0.49	3.01 ± 0.42	4.17 ± 0.50	2108.16	0.80	C3 > C2 > C1
2. Group perceived discrimination	1.59 ± 0.47	2.95 ± 0.41	4.16 ± 0.47	2302.88	0.81	C3 > C2 > C1
3. Overall perceived discrimination	1.61 ± 0.44	2.98 ± 0.32	4.16 ± 0.41	3159.00	0.86	C3 > C2 > C1

### Mental health differences across perceived discrimination subgroups

To further examine the association between latent perceived discrimination classes and mental health among college students with hearing impairment, the BCH method was used, with mental health included as a distal outcome. As shown in Table 5, mental health problems differed significantly across the three latent classes (BCH *χ*^2^ = 190.873, *p* < 0.001). Pairwise BCH comparisons showed that the low perceived discrimination group reported significantly fewer mental health problems than both the moderate and high perceived discrimination groups, and the moderate perceived discrimination group reported significantly fewer mental health problems than the high perceived discrimination group. All three pairwise comparisons remained statistically significant after Bonferroni correction for multiple comparisons (adjusted *α* = 0.017).

**Table 5:** Differences in mental health across perceived discrimination classes using the BCH method (3-4).

Variable	Low perceived discrimination (*n* = 305)			Moderate perceived discrimination (*n* = 519)			High perceived discrimination (*n* = 244)			BCH *χ*^2^	Pairwise comparisons
	*M*	SE	95% CI	*M*	SE	95% CI	*M*	SE	95% CI		
Mental health	24.33	0.33	[23.68, 24.98]	28.03	0.21	[27.62, 28.44]	30.26	0.28	[29.71, 30.81]	190.873^***^	C1 < C2; C1 < C3; C2 < C3

### Sensitivity analysis using the continuous perceived discrimination score

To further examine the association between perceived discrimination and mental health and to assess whether the LPA classification provided information beyond the continuous total score, a hierarchical multiple linear regression analysis was conducted. Gender, peer type, number of children in the family, and family economic status were entered as covariates in Model 1. Model 1 was significant, *F* (4, 1,063) = 6.567, *p* < 0.001, explaining 2.4% of the variance in mental health problems. The total perceived discrimination score was subsequently entered in Model 2. The overall model was significant, *F* (5, 1,062) = 67.219, *p* < 0.001, with an adjusted *R*^2^ of 0.237. After controlling for demographic variables, perceived discrimination was significantly and positively associated with mental health problems (*B* = 0.419, SE = 0.024, *β* = 0.473, 95% CI [0.372, 0.467], *t* = 17.389, *p* < 0.001), accounting for an additional 21.6% of the variance (Δ*R*^2^ = 0.216; Δ*F* = 302.380, *p* < 0.001). These findings were consistent with the LPA results and further suggest that the three identified classes primarily reflect severity gradations along a continuous dimension of perceived discrimination.

### Associations between demographic factors and latent class membership

A multinomial logistic regression analysis was conducted with latent class membership as the dependent variable and the high perceived discrimination group (C3) as the reference category. Variables that were significant in the univariate analyses, including gender, peer type, number of children in the family, and family economic status, were entered into the model. As shown in Table 6, compared with female students, male students had significantly lower odds of belonging to the low perceived discrimination group (OR = 0.522) and the moderate perceived discrimination group (OR = 0.695). Compared with students who reported having no close friends, those whose close friends were deaf had significantly higher odds of belonging to the low perceived discrimination group (OR = 2.463) and the moderate perceived discrimination group (OR = 1.849). In addition, compared with students from multi-child families, only-child students had significantly higher odds of belonging to the low perceived discrimination group (OR = 2.291). No significant differences in class membership were observed across family economic status categories when a monthly family income of more than RMB 10,000 was used as the reference category (all *p* > 0.05). To further account for uncertainty in latent class assignment, an R3STEP analysis was conducted in Mplus. As shown in Table 7, gender, peer type, and number of children in the family were significantly associated with latent class membership, whereas family monthly income was not significantly associated with class membership. Overall, the pattern of findings was generally consistent with that obtained from the conventional multinomial logistic regression analysis, indicating that the main associations remained stable after accounting for classification uncertainty.

**Table 6:** Multinomial logistic regression analysis of perceived discrimination class membership.

Predictor	C1 (*n* = 305) vs. C3 (*n* = 244)				C2 (*n* = 519) vs. C3 (*n* = 244)			
	OR	95% CI	*B*	*p*	OR	95% CI	*B*	*p*
Gender								
Male	0.522	0.366–0.745	−0.650	<0.001	0.695	0.504–0.958	−0.364	0.026
Peer type								
Deaf peers	2.463	1.298–4.670	0.901	0.006	1.849	1.084–3.153	0.615	0.024
Hearing peers	1.311	0.664–2.587	0.271	0.435	0.980	0.555–1.733	−0.020	0.945
Number of children in the family								
1 child	2.291	1.197–4.384	0.829	0.012	1.616	0.920–2.837	0.480	0.095
2 children	1.790	0.992–3.227	0.582	0.053	1.186	0.715–1.968	0.171	0.508
3 children	0.612	0.292–1.283	−0.490	0.194	0.782	0.430–1.422	−0.246	0.421
Family monthly income								
<RMB 2,000	1.040	0.574–1.885	0.039	0.897	1.267	0.736–2.182	0.237	0.393
RMB 2,000–5,000	1.064	0.600–1.886	0.062	0.833	1.422	0.844–2.397	0.352	0.186
RMB 5,000–10,000	0.820	0.446–1.509	−0.198	0.524	1.067	0.616–1.847	0.065	0.817

**Table 7:** R3STEP analysis of associations between demographic factors and perceived discrimination class membership.

Predictor	C2 vs. C3	*p*	C1 vs. C3	*p*	C1 vs. C2	*p*
	OR (95% CI)		OR (95% CI)		OR (95% CI)	
Gender	0.670 (0.476–0.944)	0.022	0.497 (0.346–0.713)	<0.001	0.741 (0.550–0.999)	0.049
Peer type	1.551 (1.214–1.982)	<0.001	1.657 (1.262–2.176)	<0.001	0.937 (0.730–1.203)	0.607
Number of children in the family	1.219 (1.017–1.461)	0.032	1.456 (1.193–1.777)	<0.001	0.837 (0.703–0.996)	0.045
Family economic status	1.147 (0.966–1.362)	0.118	1.090 (0.902–1.318)	0.372	1.052 (0.896–1.236)	0.531

C1, low perceived discrimination group; C2, moderate perceived discrimination group; C3, high perceived discrimination group; OR, odds ratio; CI, confidence interval. R3STEP analyses were conducted in Mplus to account for uncertainty in latent class assignment.

## Discussion

### Subgroups characterized by different levels of perceived discrimination among college students with hearing impairment

The present study identified three subgroups of college students with hearing impairment characterized by low, moderate, and high levels of perceived discrimination. Specifically, 305 students (28.6%) were classified into the low perceived discrimination group, 519 students (48.6%) into the moderate perceived discrimination group, and 244 students (22.8%) into the high perceived discrimination group. The low perceived discrimination group showed the lowest scores on both individual- and group-level perceived discrimination, whereas the high perceived discrimination group showed the highest scores across these dimensions. The moderate perceived discrimination group fell between the low and high groups, indicating an intermediate level of discrimination perception. Taken together, the three subgroups appear to represent a graded continuum of perceived discrimination severity rather than qualitatively distinct psychological configurations. Given the high correlation between individual- and group-level perceived discrimination (*r* = 0.88) and the limited number of indicators included in the LPA, these classes should not be interpreted as representing qualitatively distinct psychological patterns. Rather, they appear to reflect different levels along a common perceived discrimination severity continuum.

Across the three subgroups, individual- and group-level perceived discrimination showed a parallel pattern of change. This may be because, as individuals perceive more discrimination, their identification with their group and their perceptions of the group’s social status may correspondingly weaken, making them more likely to notice the social exclusion experienced by the group. In turn, this creates a mutually reinforcing cycle between individual- and group-level perceptions of discrimination (20), such that subjective perceptions of discrimination directed at oneself and at one’s group eventually reach a relatively consistent level. This suggests that the psychological processes underlying individual and collective perceptions of discrimination are not unidirectional, but rather intertwined and mutually reinforcing (21).

The predominance of the moderate and high perceived discrimination subgroups suggests that discrimination-related stress may be embedded in the broader educational and social experiences of students with hearing impairment. Recent qualitative evidence also shows that deaf and hard-of-hearing students in higher education continue to face barriers related to inclusion, support, social participation, and access arrangements (22). First, students with high perceived discrimination often internalize negative stereotypes imposed by others, such as being withdrawn, timid, or less capable (23), which has been associated with self-stigmatization and negative self-evaluation. This tendency may be particularly pronounced in multi-child families, where parental attention and resources are shared among siblings, making it more difficult for parents to provide sufficient emotional and autonomy support to each child (24). Such family-context differences may partly relate to the observed association between family structure and perceived discrimination subgroup membership. This may help contextualize why a relatively high proportion of college students with hearing impairment from multi-child families were classified into the high perceived discrimination group. Second, college students with hearing impairment who occupy a disadvantaged or “othered” social position often experience alienation from both hearing individuals and other persons with hearing impairment (25). They may struggle to integrate into the social circles of hearing students while also lacking a strong sense of identification with their in-group, which may co-occur with lower core self-evaluation and higher perceived discrimination. In addition, male college students with hearing impairment may place greater importance on competence in social interactions; consequently, communication barriers may be more readily experienced as a form of discrimination implying incompetence. Because they may be less skilled in interpersonal communication and more likely to make internal attributions (7), they may respond to discrimination-related experiences through avoidance or concealment rather than active expression, which may further maintain or intensify their perceived discrimination (26). Similarly, when their peers are hearing individuals, college students with hearing impairment may perceive themselves as disadvantaged in social comparison, experience lower friendship quality, and become increasingly aware of the differences between themselves and surrounding groups, thereby developing anxiety, inferiority, and a reduced sense of control (27), which may further reinforce perceived discrimination.

### Mental health differences across perceived discrimination severity subgroups

The findings showed that perceived discrimination was significantly positively associated with mental health problems, consistent with previous evidence linking perceived discrimination to poorer psychological well-being (28). Among the three groups, students in the high perceived discrimination group reported the highest level of mental health problems. Previous research has linked perceived discrimination with lower perceived control, greater helplessness, depressive and anxiety symptoms, and difficulties in psychological adjustment (21, 29, 30). These factors may represent possible psychosocial processes underlying the observed association; however, they were not directly examined in the present study and should therefore be interpreted cautiously. Previous research has also associated everyday discrimination with lower self-control, greater reliance on expressive suppression, and higher depressive symptoms (31). Such findings suggest that self-regulatory and emotion-regulation processes may be relevant to the association between perceived discrimination and psychological distress. However, these mechanisms were not directly assessed in the present study. Similarly, although adaptive coping and stronger self-identification may be relevant protective factors, the present cross-sectional data do not allow conclusions regarding the mechanisms underlying differences between the low, moderate, and high perceived discrimination groups.

Students with moderate perceived discrimination often remain in a subtle state of perceiving covert discrimination, such as experiencing slight neglect, unintentional indifference, or institutional exclusion. Although these forms of discrimination may not be accompanied by immediately apparent psychological difficulties, the present findings indicate that moderate perceived discrimination is nevertheless associated with significantly greater mental health problems than low perceived discrimination. Given the relatively subtle nature of moderate perceived discrimination, early identification and psychological support may be particularly important for this group.

## Practical implications

### Strengthen screening for perceived discrimination and psychological risk among students with hearing impairment

Perceived discrimination was significantly associated with mental health problems among college students with hearing impairment in the present study and may therefore serve as a useful psychosocial indicator in mental health screening. Standardized perceived discrimination scales should therefore be incorporated into routine mental health screening systems for this population and used as an important basis for assessment and intervention. University counseling centers and departmental support units should establish screening mechanisms specifically targeting perceived discrimination among students with hearing impairment, using multiple sources of information, including student self-reports, peer ratings, and teacher evaluations, to conduct regular and systematic assessments. Assessment content should cover both individual- and group-level perceived discrimination and should extend to quality of campus life, learning climate, and teacher-student interactions, so as to comprehensively capture the manifestations and levels of perceived discrimination and identify high-risk groups at an early stage.

Given that male students account for a relatively large proportion of college students with hearing impairment, and given the gender differences observed in perceived discrimination subgroup membership in this study, it is advisable to incorporate a gender-sensitive perspective into screening procedures. Gender-specific versions of the assessment may be developed to evaluate differences in the manifestations of perceived discrimination and their associations with mental health among male and female students, thereby enabling more precise risk identification and intervention. Future research could explore whether digital assessment approaches, such as ecological momentary assessment or mobile sensing, may facilitate the timely identification of changes in perceived discrimination and psychological distress among students with hearing impairment (32).

### Potential implications for tiered psychological support

To improve the precision of psychological interventions, stratified intervention strategies should be developed based on the three perceived discrimination severity subgroups. For the low and moderate perceived discrimination groups, the primary focus should be the prevention of psychological problems. Peer-support programs, peer mentoring, and structured group-based skills training may be used to enhance emotional support, stress coping, and peer interaction, thereby helping prevent the development of psychological problems (33), while interactive methods such as music therapy and psychodrama may be employed to enhance social adaptation (34). At the same time, universities should ensure fairness in resource allocation by providing students with hearing impairment with equal, or where appropriate greater, access to learning opportunities, merit-based recognition, and institutional support, thereby alleviating negative emotions caused by relative deprivation (35) and weakening the tendency toward heightened perceived discrimination resulting from unequal resource distribution.

Students with high levels of perceived discrimination may warrant particular attention in university mental health services. Although specific interventions were not evaluated in the present study, previous research suggests that targeted psychological approaches may have potential value for supporting individuals experiencing discrimination-related psychological difficulties (36). Future studies should evaluate the effectiveness of such interventions among college students with hearing impairment before specific intervention strategies can be recommended.

### Improve family and peer support for mental health

Social support is an important external resource for alleviating perceived discrimination and promoting mental health (37). In addition to formal sources of support from schools and society, informal support from family members, friends, and neighbors also plays a crucial role in the psychological development of students with hearing impairment (38). The present study showed that family structure and peer type were significantly associated with perceived discrimination subgroup membership among college students with hearing impairment. It is therefore necessary to build a multidimensional support system linking families, universities, and peers.

University counseling centers should take the lead in establishing an “inclusive growth support program” that systematically connects hearing students, students with hearing impairment, and their parents, thereby fostering an inclusive campus environment. On the one hand, online platforms incorporating accessibility technologies, such as sign-language recognition and speech-to-text and text-to-speech tools (39), may facilitate communication among students with hearing impairment, their parents, and peers, thereby enhancing mutual understanding and strengthening social connections. On the other hand, offline activities such as situational simulations and role-model sharing may be combined with regular online micro-sharing sessions and themed discussions to improve the communication skills and support capacities of families and peers, maintain the continuity and everyday nature of psychological support, and facilitate the integration of family support, peer support, and professional school-based services. In this way, perceived discrimination may be effectively buffered, while students’ sense of belonging and psychological resilience may be enhanced.

## Conclusion and limitations

Using LPA, this study identified three subgroups of college students with hearing impairment characterized by low, moderate, and high levels of perceived discrimination. Individual- and group-level perceived discrimination changed in parallel across the three subgroups, indicating that the identified classes primarily represented differences in the overall severity of perceived discrimination rather than qualitatively distinct configurations. Membership in these severity-based subgroups was significantly associated with mental health problems, with students in the high perceived discrimination group reporting the most severe mental health problems. These findings suggest that a person-centered approach may complement variable-centered analyses by identifying groups of students reporting different levels of perceived discrimination and psychological distress, thereby providing a potential basis for targeted screening and tiered psychological support.

Nevertheless, this study has several limitations. First, the cross-sectional and self-report design limits causal inference and may introduce shared method variance. Although higher perceived discrimination was associated with greater mental health problems, the temporal and causal direction of this association cannot be determined, and reciprocal relationships are possible. In addition, residual confounding by socioeconomic circumstances, social support, communication access, hearing-device use, and pre-existing psychological conditions cannot be excluded because these factors were not comprehensively assessed or controlled in the present analyses. Moreover, all variables were assessed by the same participants at the same time point. Although the single-factor CFA indicated that the covariance structure was not adequately represented by a single common factor, shared method variance cannot be ruled out. Future studies should use longitudinal, multi-informant, and objective or behavioral measures to clarify temporal and potentially bidirectional associations.

Second, the LPA was based on six item-level indicators derived from two highly correlated dimensions of perceived discrimination—individual- and group-level perceived discrimination. Consequently, the identified classes primarily reflected differences in overall severity rather than qualitatively distinct psychological configurations. Future studies could incorporate a broader range of discrimination indicators, such as interpersonal, institutional, and context-specific experiences, to examine whether more differentiated patterns emerge.

Third, although the sample was relatively large and geographically diverse, participants were recruited using purposive sampling from 21 Chinese universities that admit students with hearing impairment through separate entrance examinations for students with disabilities. Therefore, the sample should not be considered nationally representative of college students with hearing impairment in China. In particular, the findings may not generalize to students with hearing impairment admitted through mainstream university admission pathways, individuals with hearing impairment outside higher education, or populations in other cultural contexts. Future studies should recruit more diverse samples across different admission pathways, educational settings, and sociocultural contexts. In addition, participants were nested within 21 universities, with unequal numbers of participants across institutions, but potential institution-level clustering was not accounted for in the present analyses. Consequently, un-modeled between-institution variation may have affected the estimated standard errors, confidence intervals, and statistical significance of the observed associations. The corresponding inferential results should therefore be interpreted with appropriate caution. Future studies should consider multilevel or cluster-adjusted analyses when institution-level identifiers and sufficiently large within-institution samples are available.

## Data Availability

The datasets generated and analyzed during the current study are available from the corresponding author upon reasonable request, subject to ethical approval and applicable data-protection requirements.
